# Pernicious Vomiting of Pregnancy and Its Treatment with Electricity*Read before the Brazos Valley Medical Association at Caldwell, Texas, May 8, 1900.

**Published:** 1900-08

**Authors:** J. P. Oliver

**Affiliations:** Caldwell, Texas


For the Texas Medical Journal.

### Pernicious Vomiting of Pregnancy and its Treatment with Electricity.*

DR. J. P. OLIVER, M. D., CALDWELL, TEXAS.

In using the word pernicious in this article, it is intended to convey the same idea that is expressed when we speak of pernicious forms of malarial and other diseases to which the word is applied, simply an intensification of the cause or causes entering into the case. I shall not attempt to discuss conception in any of its accepted theories, but rather this peculiar phase of it. Nausea, vomiting and hypersecretion of saliva, together with perversion of taste and smell, and other nervous phenomena, are usually present in all normal pregnancies in some degree.

Perhaps it would be opportune for me to give you a symposium of the views of the authorities upon this subject, that we may get the data of said authorities up to date, and apply them with our experiences, and see how the practical points agree. Horowitz says that, "the pernicious vomiting of pregnancy, which is worse at the very outset, soon leads to complete intolerance of all possible ingesta, as well as constant retching when the stomach is empty." He makes two periods to this affection. In the first we have nausea and anorexia, ptyalism hyperemia, irritability and apathy.

Vomiting in this period is associated with the act of taking nour-

---

*Read before the Brazos Valley Medical Association at Caldwell, Texas, May 8, 1900.

ishment, or, if you please, the mere suggestion of taking nourishment, or the scent of anything along this line, as was the case in the last patient I saw. In the second period, vomiting is continuous. Much suffering results from emesis with empty stomach. There is general appearance of inanition. The mouth is dry, breath has a repulsive odor, thirst is complained of, the bowels are obstinately constipated. Death said to occur under coma. The traditional view is reflex neurosis from gravid uterus.

Tyler Smith and Schroeder explained the reflex by hyperdistension of the womb; polyamnois, twins, etc.; Graily Hewitt, by malpositions of the uterus, such as anteflexion, retroflexion and retroversions. This accords with my experience. Add to the above conditions laceration of the cervix with cicatricial tissue, cervicitis metro, and endometritis, and you can have any reflex symptom almost imaginable. In fact, I have had more trouble and less satisfactory results in the treatment of retroversions and prolapsed ovaries, including cases of neurasthenia, than any other class of cases. We all know how stubborn some of these cases are, and how slow and unavailing our treatment is, because, with retroversions, ranging from forty-five to one hundred and thirty-five degrees, some of them of long standing, dating back to former pelvic troubles, we invariably have obstructed venous circulation of the uterine mucosa, and its concomitant results; that is, cervicitis, endocervicitis, metritis and endometritis. So, when conception occurs in these cases we not only have the malposition to contend with, but the conditions above mentioned. Practically, I know of no trouble giving such poor results as cases of retroversion of long standing, whether they be complicated with morbid growths or not, and you may have any form of reflex symptoms, from the normal nausea and vomiting of pregnancy to the pernicious form, of which I am speaking.

I am aware that some practitioners claim good results from the use of pessaries. This symptom of treatment, I am proud to say, has seen its best day, and all that can be accomplished with pessaries can be done by the knee and elbow position, with a glycerole of cotton well placed in the posterior cul-de-sac of the vagina, and some favor Alexander's operation on the sound ligaments of the uterus. Of the latter I cannot speak advisedly, as I have never performed it, nor do I ever expect to for retroversion. Horrowitz says, that the [cause of] hyperemesis, if local, ought to be found in metritis.

Julien, in 1887, went a little further, and said that the whole trouble was peritoneal in origin. There are some women, he said, in whom the serous covering of the womb will not expand. Isolated cases in practice appear to give support to all these views. Holladay blamed the ovary; thought the growth of the corpus luteum caused the reflex trouble.

A number of authors have noted pernicious vomiting in extra-uterine pregnancy. In the study of these cases, we are confronted with another question of some magnitude, which demands some attention. Does pernicious vomiting ever occur in conditions other than pregnancy? I shall answer this in the affirmative. because we all have lost patients in pernicious forms of malarial fever when the vomiting continued to the end; besides, in gastric ulcer and malignant forms of diseases of the stomach, uncontrollable vomiting is frequently a prominent symptom.

Austrebe goes over all the varied hypotheses as to the nature of hyperemesis, to see if any of them could explain the action of cocaine. He rejects them all in turn, so that by exclusion there is nothing left to accuse except the medulla oblongata, in which is seated the center of emesis. Increased excitability of this center is able to explain hyperemesis. He claims that cocaine benefits not only the nerve centers in the medulla, but the peripheral nerve distribution in the cardiac orifice in the stomach. This author uses a strong solution of cocaine, and uses it in heroic doses.

It will be observed from the foregoing remarks that the diagnosis of pernicious vomiting of pregnancy is no easy matter, as the standard authorities from all the schools of medicine have viewed it from different standpoints. In the majority of the fatal cases of alleged hyperemesis due to pregnancy reported by American, French and English authors, there is a notable absence of reliable records of post-mortem examinations. In the few cases collected by the Germans, on the other hand, the diagnosis during life has almost invariably been confirmed or negatived by exact investigation of the dead body. The diffenential diagnosis between the pernicious vomiting of pregnancy and other causes independent of gestation, demands very colse observation of the physician. I can call to mind only four cases in my practice of thirty years. Two of them had albuminuria and died, as the vomiting was complicated and uncontrollable in two, and each died without post-mortem verification. I am not certain that the kidney trouble was not the cause of death in both. The other two recovered. One aborted in about five weeks from the initial attack; came very near dying from hemorrhage. Two factors were instrumental in saving the life of this patient. First, nourishment by enema and persistent use of oleaginous ointments. The latter, I believe, exercised some influence on nutrition. The other factor was getting rid of the fœtus at about the third month of gestation.

My fourth case will embrace the following report of a case treated by Dr. McLean and myself in this town.

Mrs. J.; family history that of pulmonary tuberculosis, several of the family on one side having died of it. Personal history: Married about 23 or 24 years old; mother of two children, and about two or two and a half months advanced in the third gestation. Patient stated that she had suffered nearly all the time since conception had occurred, from extreme nausea, vomiting and retching, and had retained but very little on the stomach of any meal she had eaten during this time. Dr. McLean was called to see her, and after using all the usual remedies without avail, including the much vaunted cocaine, suggested that I be called in consultation. On my arrival, the foregoing statements were not only confirmed, but I found the woman very much emaciated, low form of fever, constipated bowels, urine albuminous and containing considerable amount of uric acid and urea, and the top of one lung involved, with persistent hyperemesis; no rest night nor day only for a few moments at a time. Nor did the stomach retain any amount of nourishment to do any good. At this juncture of the case we were somewhat in a dilemma as to what course to pursue. Husband and wife insisting on abortion, and pressing it, for the safety of the wife, Dr. McLean having applied a strong solution of silver to the os and the usual remedies by the mouth, you can readly appreciate our situation. The life of our patient threatened with inanition, and all the functions apparently locked, and to add fuel to the fire, our patient not only had the tuberculosis diathesis, but an actual involvement of one lung. To perform abortion we thought hazardous in the extreme, so I suggested that we use an anæsthetic, dilate the os an inch or more, apply silver solution, use morphia hypodermatically, nourish by enema and await results, promising, after stating to the husband the great risk to run in producing abortion in her case on account of her family history, that if these remedies did not relieve her, that he would abort, provided a third physician's consent was obtained. The pressure was very great on us to terminate gestation. In the meantime, our patient continued to have hyperemesis night and day, only when narcotized with morphia. Having exhausted the last efforts without any relief whatever, only temporary to our patient, I then suggested that we use electricity twice daily with the Farradic current, and continue nutrient enema and hypodermic morphia and atropia until the electricity could have time to produce its effects. We alternated with the negative and positive up and down the entire spinal column and the stomach, Farradized the pneumogastrics as best we could, continued enemas of milk punch, malted milk and soups twice daily for about ten days. To our great surprise, our patient soon began to improve, and in six or eight days could take light diet by the stomach. The hyperemesis gradually gave way and our patient took the train and left for a higher altitude, apparently cured of the persistent hyperemesis.

This case was one pre-eminently of neurasthenia, in my opinion. My theory about the benefit the electricity did in the case is this: It will be remembered that it is generally claimed that the pernicious hyperemesis is a reflex neurosis, either central or peripheral in creation. In this case possibly the diathesis exercised some influence. There is a very strong possibility, if not a probability, that there was tuberculous irritation somewhere either about the nerve center for emesis, or in the serous covering of the uterus, which was controlled by the electricity. Evidently it was the remedial agent that gave relief. For instance, when the hypodermic medicine was begun hyperesthesia was so great that we would have to give an anæsthetic to some extent before using it. As there was no malposition of the uterus, nor its appendages perceptible, those can be excluded from the peripheral cause, and brings us to the nervous system and the diathesis as the chief factors in this case which the electricity controlled and the nutrient enemas sustained.

